# Metabolic signatures differentiate ovarian from colon cancer cell lines

**DOI:** 10.1186/s12967-015-0576-z

**Published:** 2015-07-14

**Authors:** Anna Halama, Bella S Guerrouahen, Jennifer Pasquier, Ilhem Diboun, Edward D Karoly, Karsten Suhre, Arash Rafii

**Affiliations:** Department of Physiology and Biophysics, Weill Cornell Medical College in Qatar, Qatar-Foundation, P.O. Box 24144, Doha, Qatar; Stem Cell and Microenvironment Laboratory, Weill Cornell Medical College in Qatar, Education City, Qatar Foundation, Doha, Qatar; Department of Genetic Medicine, Weill Cornell Medical College, New York, NY 10065 USA; Experimental Biology Division-Research, Sidra Medical and Research Center, PO Box 26999, Doha, Qatar; Metabolon, Inc., Durham, NC 27713 USA; Institute of Bioinformatics and Systems Biology, Helmholtz Zentrum München, German Research Center for Environmental Health, Neuherberg, Germany; Department of Genetic Medicine and Obstetrics and Gynecology, Weill Cornell Medical College, Stem Cell and Microenvironment Laboratory, Weill Cornell Medical College in Qatar, Qatar-Foundation, P.O. Box 24144, Doha, Qatar

## Abstract

**Background:**

In this era of precision medicine, the deep and comprehensive characterization of tumor phenotypes will lead to therapeutic strategies beyond classical factors such as primary sites or anatomical staging. Recently, “-omics” approached have enlightened our knowledge of tumor biology. Such approaches have been extensively implemented in order to provide biomarkers for monitoring of the disease as well as to improve readouts of therapeutic impact. The application of metabolomics to the study of cancer is especially beneficial, since it reflects the biochemical consequences of many cancer type-specific pathophysiological processes. Here, we characterize metabolic profiles of colon and ovarian cancer cell lines to provide broader insight into differentiating metabolic processes for prospective drug development and clinical screening.

**Methods:**

We applied non-targeted metabolomics-based mass spectroscopy combined with ultrahigh-performance liquid chromatography and gas chromatography for the metabolic phenotyping of four cancer cell lines: two from colon cancer (HCT15, HCT116) and two from ovarian cancer (OVCAR3, SKOV3). We used the MetaP server for statistical data analysis.

**Results:**

A total of 225 metabolites were detected in all four cell lines; 67 of these molecules significantly discriminated colon cancer from ovarian cancer cells. Metabolic signatures revealed in our study suggest elevated tricarboxylic acid cycle and lipid metabolism in ovarian cancer cell lines, as well as increased β-oxidation and urea cycle metabolism in colon cancer cell lines.

**Conclusions:**

Our study provides a panel of distinct metabolic fingerprints between colon and ovarian cancer cell lines. These may serve as potential drug targets, and now can be evaluated further in primary cells, biofluids, and tissue samples for biomarker purposes.

**Electronic supplementary material:**

The online version of this article (doi:10.1186/s12967-015-0576-z) contains supplementary material, which is available to authorized users.

## Background

The treatment of complex diseases like cancer still remains a major challenge, both for patients and for the healthcare system. Better characterization of tumor identity through a comprehensive “-omics” approach has modified paradigms in translational cancer research. By combining several analyses, major consortiums have been driven to describe tumor-specific landscapes. Transcriptomic studies have led to the definition of several tumor-specific subtypes, leading to optimal staging as well as tailored treatment. Finally, the characterization of epigenetic changes has also recently informed clinicians about tumor plasticity as a mechanism that supports therapeutic escape. Aside from the large body of clinical work, most of these novel techniques have been optimized using model cancer cell lines. The use of model cell lines has clearly culminated in the cancer cell line encyclopedia (CCLE) project, in which multiple cancer cell lines have been characterized in detail using several “-omics” platforms.

Metabolomics is the study of the small molecule composition (metabolites <2,000 Da) in bio-fluids, tissue samples, and cell lines. By measuring the consequences of all changes in gene expression, protein abundance, and environmental influence, metabolomics has been recognized as the “-omics” technology that provides readouts that are closest to the clinical endpoint [1]. Metabolomics approaches based on high-throughput technologies, mostly including mass spectrometry [e.g., liquid chromatography–mass spectrometry (LC–MS), ultrahigh-performance liquid chromatography–mass spectrometry (UPLC–MS), or gas chromatography–mass spectrometry (GC–MS) or nuclear magnetic resonance spectroscopy (NMR)] tools, have recently become the main strategies for identifying novel biomarkers and elucidating the etiology of complex diseases, foremost diabetes [2] and cancer [3]. There are still several open questions in the field of complex disorders that can be addressed by applying metabolomics. For instance, it has been reported that the ovary is a site of metastasis for several cancer types, and particularly colorectal cancer [4]. Nevertheless, differentiation between primary ovarian tumors and ovarian metastases that originate from primary colon tumors is difficult with available radiological approaches, and can remain confusing after histopathological analysis. Assays that enable clear differentiation between primary ovarian tumor and ovarian metastasis from tissue or biofluids samples could strongly support correct diagnosis and patients’ outcomes. This issue has already been addressed using genomics, proteomics, and tissue array profiling approaches, and enables the determination of tissue-specific patterns [5]. We believe that determining which metabolic markers present in biofluids are able to differentiate between primary ovarian tumor and ovarian metastasis from colon tumors could improve diagnostic capability.

Metabolomics has already been used to identify biomarkers of ovarian and colon carcinomas in plasma [6, 7] and tissue samples [8, 9]; however, these reports focus on biomarkers that differentiate cases from controls, rather than cancers from different origins. Additionally, human biofluids are not an optimal matrix for study when attempting to identify and understand metabolic patterns from two different cancer types, because several factors (e.g., age, gender, or daily habits) might have a strong impact on whole-body metabolism and overshadow patterns of interest. Metabolic studies in cell culture are highly valuable [10] to identify functional biomarkers that represent cellular processes [11–13] or cancer cell lines’ individuality [12, 14, 15], and are essential for a comprehensive understanding of cell biology and to complement clinical studies [10].

The main goal of this study was to determine the metabolic signatures of colon and ovarian cancer cell lines, which might serve several purposes. First, we endeavored to determine the metabolic signatures of ovarian and colon cancer cell lines, which could be evaluated in greater detail to determine metabolic fingerprints for cell identity purposes. The identified metabolic signatures and pathways will provide insight into the pathophysiology of ovarian and colon cancer cell lines. Second, we attempted to identify metabolic processes and pathways that distinguish ovarian and colon carcinomas that might be targetable to control neoplastic disease, with potential clinical applications. We applied non-targeted metabolomics to profile four different cell lines from colorectal (HCT15, HCT116) and ovarian (OVCAR3, SKOV3). We identified metabolic signatures and pathways that clearly demonstrated differences between the two tumor types.

## Methods

### Cell culture

Established cancer cell lines were obtained from the American Type Culture Collection (ATCC, Manassas, VA, USA). Upon receipt, cells were expanded; at <10 passages, aliquots of cells were stored frozen in liquid nitrogen. Cells from an aliquot were kept in culture for <2 months. Human ovarian adenocarcinoma cell lines SKOV3 (HTB-77) and OVCAR3 (HTB-161) were grown in Dulbecco’s modified Eagle medium high glucose (Hyclone, Thermo Scientific) supplemented with 10% fetal bovine serum (FBS; Hyclone, Thermo Scientific), 1% penicillin–streptomycin solution (Sigma), 2 mM l-glutamine (Sigma), 1× non-essential amino acids (Hyclone, Thermo Scientific). The human colorectal adenocarcinoma cell lines HCT15 (ATCC CCL 225) and HCT116 (CCL247) were maintained in McCoy’s 5A medium supplemented with 10% heat-inactivated FBS, 2 mM l-glutamine, and 1% penicillin–streptomycin solution. For the experiments, cells were trypsinized and washed with PBS, and all cell lines were cultivated in M199 medium to avoid the effect of culture milieu on metabolic profile. After incubation overnight, cells were thoroughly washed, medium was changed, and cells were cultivated in fresh medium for the next 24 h. Once cells reached 80% confluence (approximately 48 h after cell were seeded), samples were collected by trypsinization. One million cells were counted and washed with PBS; cell pellets were frozen at −80°C. Ten replicates were prepared for each cell line.

### Sample preparation for metabolic profiling

Frozen pellets were sent to Metabolon Inc. (Durham, NC, USA) for metabolic profiling, where all procedures were performed according to Metabolon’s standard protocols. All samples were maintained at −80°C after processing with the MicroLab STAR^®^ system from Hamilton Company. In a first step recovery standards were added for quality control (QC) purposes. Samples were extracted with series of organic and aqueous solvents to remove the protein fraction, allowing the maximum recovery of small molecules (metabolites). Each sample extract was split into equal parts for GC and LC analysis. After the removal of organic solvent, samples were frozen and vacuum-dried.

### Metabolite measurements with UPLC–MS

Each sample extract designated for LC–MS analysis was split into two aliquots, dried, and reconstituted in acidic or basic LC-compatible solvents, each of which contained eight or more injection standards at fixed concentrations to ensure injection and chromatographic consistency. The analysis of LC/MS samples performed on the platform using a Waters ACQUITY UPLC system and a Thermo Fisher Scientific Orbitrap Elite high resolution/accurate mass spectrometer, which was composed of a heated electrospray ionization (HESI) source and an orbitrap mass analyzer operating at 30,000 mass resolution. Samples were analyzed using acidic positive ion optimized conditions (one aliquot) and basic negative ion optimized conditions (second aliquot) in two independent injections on separate dedicated columns. Extracts reconstituted in acidic conditions were gradient eluted using water and methanol containing 0.1% formic acid, while the basic extracts used water/methanol containing 6.5 mM ammonium bicarbonate. The MS analysis alternated between MS and data-dependent MS^2^ scans using dynamic exclusion.

### Metabolite measurements with GC–MS

Samples assigned for GC–MS analysis were re-dried under vacuum for at least 24 h prior to derivatization. The derivatization process was performed using *N*,*O*-Bis(trimethylsilyl)trifluoroacetamide (BSTFA) under dried nitrogen conditions. The GC column was 5% phenyl, and the temperature ramp was from 40 to 300°C over a 16-min span. The measurements were performed on a Thermo-Finnigan Trace DSQ fast-scanning single-quadrupole mass spectrometer instrument using electron impact ionization, which was tuned and calibrated daily for mass resolution and mass accuracy.

### Metabolite identification

Peaks obtained after measurements were identified using Metabolon’s propriety peak integration software, and component parts were stored in a separated and specifically designed complex data structure. Metabolites were identified by comparing the obtained data to the library entries of purified standards or unknown recurrent entities. On the date of data evaluation, more than 3,000 commercially available purified standard compounds were acquired and registered in the Laboratory Information Management System (LIMS) for distribution to both LC and GC platforms for the determination of their analytical characteristics. The combination of chromatographic properties and mass spectra provided an indication of a match to the specific compound or isobaric entity. To achieve the highest data quality for statistical analysis, series of QC and curation processes were conducted. This system enables accurate and consistent identification of true chemical entities with simultaneous removal of system artifacts, mis-assignments, and background noise. Library matches for each compound were checked for each sample, and manually corrected if necessary.

Because sample measurements were obtained during a 5-day period, data were normalized to correct variations resulting from inter-day tuning differences in the instrument. Essentially, each compound was corrected in a run-day by registering the medians to equal one, proportionally normalizing each data point.

### Metabolomics data

In total, we identified 533 metabolites, including 435 metabolites of known identity and 98 molecules of unknown identity. The median number of metabolites detected in any single sample in the whole data set was 405, the median number of metabolites detected in a single sample per cell line were as follows: HCT15, 372; HCT116, 391; OVCAR, 438; SKOV3, 467. Metabolites with >20% missing (N = 308) values were removed from the data set, and metabolites with <20% missing values (N = 225) were imputed to the average in the group.

### Statistical data analysis

Statistical data analysis was performed using the web-based tool *meta*P-server at the Helmholtz Center Munich (http://metabolomics.helmholtz-muenchen.de/metap3), which provides automated and standardized data analysis for quantitative metabolomics data [16]. The server calculates general statistical measures for the metabolite quantifications, including mean, median, and, standard deviation in relation to the mean; it also provides principal component analysis (PCA), hypothesis tests, and correlation analysis [16]. For visualization, *meta*P-server creates PCA plots, bar plots, and box plots [16].

Metabolic data was normalized against cell number (1 × 10^6^ cells). PCA was applied to identify outliers. Two outliers in the SKOV3 cell line were identified and removed, possibly caused by some non-traceable experimental or technical errors (Additional file 1: Figure S1).

The non-parametric Kruskal–Wallis test for multi-class categorical phenotypes was applied to test the association of metabolite concentration with cell line. We used the Mann–Whitney, non-parametric hypothesis test for two-class phenotype. Metabolites that enable differentiation between cancer types were identified based on the following criteria: significant difference between ovarian and colon cell lines (p < 2.22 × 10^−4^ for comparison HCT116 + HCT15 vs. OVCAR3 + SCOV3), but no differences within groups of cell lines from the same origin, (p > 2.22 × 10^−4^ for HCT116 vs. HCT15 and OVCAR3 vs. SCOV3).

We adopted the most conservative approach to inferring significant differences between cancer types; statistical significance between cancer types and between cell lines was inferred using stringent Bonferroni correction to account for testing 225 metabolites (p < 0.05/225 = 2.22 × 10^−4^). The full data set and *meta*P analysis are freely and fully available at the following websites: ovarian vs. colon: http://metap.helmholtz-muenchen.de/metap3/run.cgi?ID=1415435214597630; HTC15 vs. HTC116: http://metap.helmholtz-muenchen.de/metap3/run.cgi?ID=1415362867314825; SKOV3 vs. OVCAR: http://metap.helmholtz-muenchen.de/metap3/run.cgi?ID=1415431362747244; all cell lines HTC15, HTC116, OVCAR3, and SKOV3: http://metap.helmholtz-muenchen.de/metap3/run.cgi?ID=141536220398464.

Hierarchical clustering (HCL) was performed using the MultiExperiment Viewer (MeV) v. 4.9 software [17], based on log-scaled and z-scored data, Pearson correlation as a distance measure, and average linkage clustering.

## Results

### Overall metabolic signatures of colon and ovarian cancer cell lines

**Figure 1:**
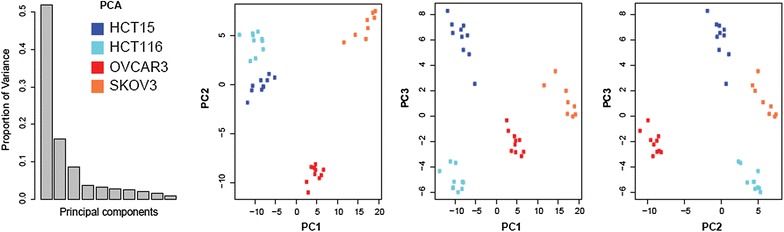
Metabolic diversity of the examined cell lines. The PCA score plots demonstrate distinct clustering of HCT15 (*dark blue*), HCT116 (*light blue*), OVCAR3 (*red*), and SKOV3 (*orange*). Clear separation of cell lines was observed. The number of *dots* corresponds to the number of replicates. Data was analyzed using the *meta*P server [16] and can be accessed interactively at http://metap.helmholtz-muenchen.de/metap3/run.cgi?ID=141536220398464.

We applied non-targeted metabolomics in order to determine the metabolic signatures of four widely used cancer cell lines, two from colorectal carcinoma (HCT15 and HTC116) and two from ovarian carcinoma (OVCAR3 and SKOV3). The obtained dataset resulted in 225 individual metabolites, which in turn were used in the analysis (see Additional file 2: Table S1 for a full list of all metabolites). PCA revealed clear separation between the four cell lines, with a tight clustering of biological replicates (Figure 1), confirming their strong metabolic diversity.

**Figure 2:**
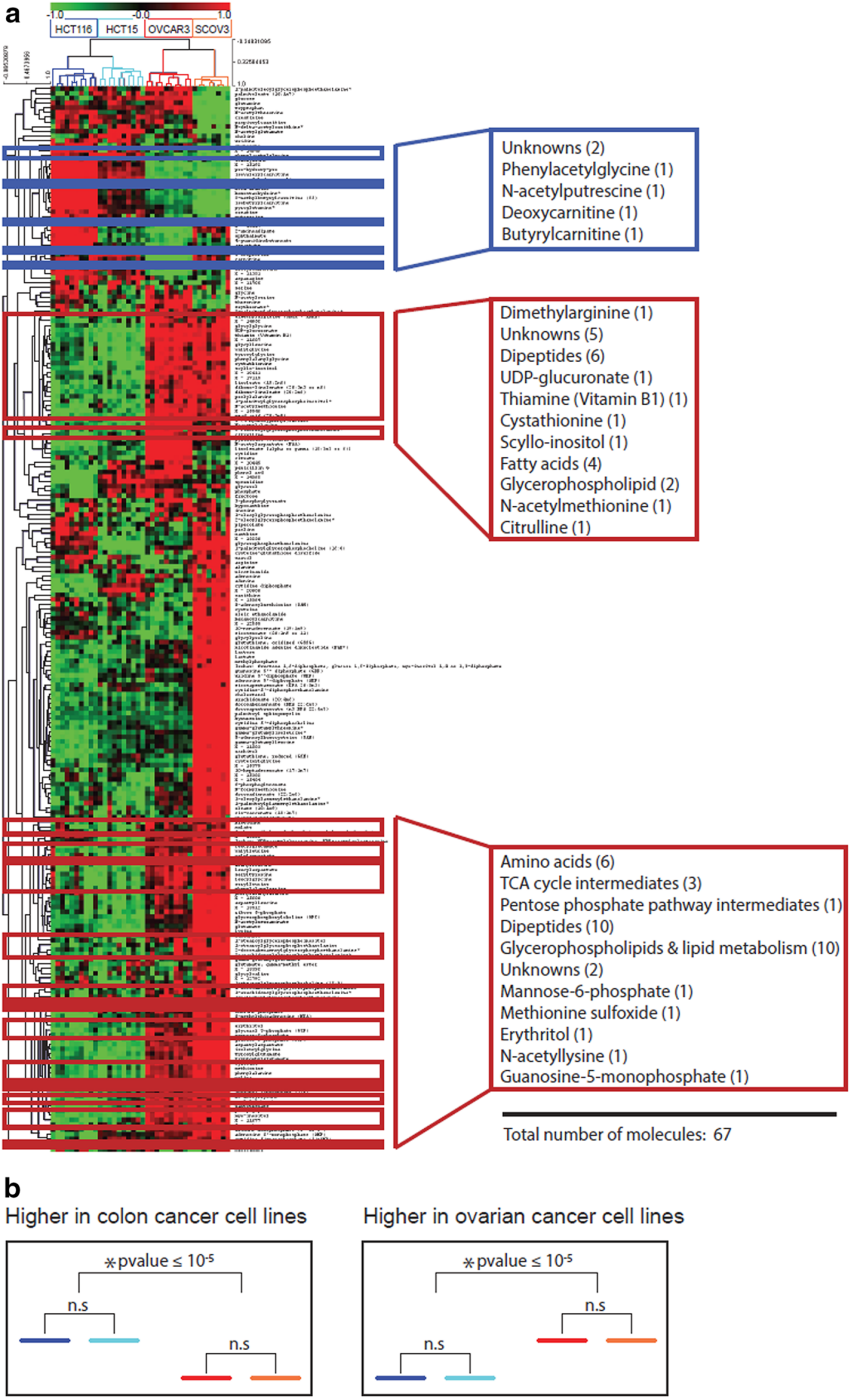
A heat map of the metabolite profiles. **a** *Colour coding* displays differences between cell lines: *green* indicates lower levels and *red* indicates higher levels of metabolite intensity (z-scored data). Samples are indicated by *blue* color gradation for colon cancer cell lines (HCT15, *dark blue*; HCT116, *light blue*), and *red* and *orange* color gradation for ovarian cancer cell lines (OVCAR3, *red*; SKOV3, *orange*). Metabolites that significantly differentiate colon from ovarian cancer cell lines are framed. The *blue frame* indicates metabolites detected at higher levels in colon cancer cell lines; the *red frames* indicate metabolites observed at higher levels in ovarian cancer cell lines. **b** Exemplified model of patterns investigated across the metabolites for identification of molecules differentiating colon from ovarian carcinomas.

To provide broader insight into the data set, we applied bidirectional hierarchical cluster analysis to all 225 metabolites (Figure 2a). All samples were split at the highest level by cancer type, and at a second level by cell line, with biological replicates clustering closely together. Metabolites clustered into four major groups according to their patterns; these groups exhibited alterations specific to: each cell line, cell lines from different origins as well as cell lines from a common origin. Examples of cell line-specific metabolites are presented as box plots in Additional file 3: Figure S2. Comparison of the metabolic profiles of cell lines from the same origin (OVCAR3 vs. SKOV3 and HCT15 vs. HCT116) resulted in the identification of 105 metabolites that significantly differentiated OVCAR3 from SKOV3 (Additional file 4: Table S2) and 48 metabolites that significantly differentiated HCT15 from HCT116 (Additional file 5: Table S3).

### Metabolites that differentiated colon from ovarian cancer cell lines

To identify metabolites that are specifically related to cancer cells’ origin (colon vs. ovary), we identified molecules that differed significantly between ovarian and colon cell lines (p < 2.22 × 10^−4^ for comparison of HCT116 + HCT15 vs. OVCAR3 + SCOV3), but not within groups of cell lines from a common origin (p > 2.22 × 10^−4^ for HCT116 vs. HCT15 and OVCAR3 vs. SCOV3) (Figure 2b). We identified 67 metabolites that follow this pattern (Table 1). Because we compared metabolic signatures of ovarian and colon cancer cell lines, the term significantly higher or lower, in this manuscript, refers to the significant difference in metabolic signature when comparing one group versus another.

**Table 1:** Metabolites that distinguish colon cancer cell lines from ovarian cancer cell lines

Metabolic group/metabolites	Ovarian-colon	Levels in ovary	HCT15_HCT116	SKOV3_OVCAR3
	p value		p value	p value
**Amino acid metabolism**				
Citrulline	5.48 × 10^−7^	Higher	0.0433	0.0003
Cystathionine	5.96 × 10^−11^	Higher	0.1051	0.0676
Dimethylarginine (SDMA + ADMA)	2.08 × 10^−7^	Higher	0.2176	0.0545
Histidine	1.61 × 10^−7^	Higher	0.1230	0.6965
Isoleucine	1.13 × 10^−9^	Higher	0.2475	0.0003
Methionine	5.96 × 10^−11^	Higher	0.7959	0.0343
Methionine sulfoxide	1.79 × 10^−9^	Higher	0.0011	0.0021
*N*-acetylmethionine	1.79 × 10^−9^	Higher	0.4813	1.0000
*N*-acetylputrescine	1.54 × 10^−7^	Lower	0.2176	0.2133
N6-acetyllysine	4.17 × 10^−10^	Higher	0.0015	0.0676
Phenylacetylglycine	1.52 × 10^−7^	Lower	0.1431	0.0043
Phenylalanine	5.96 × 10^−11^	Higher	0.5288	0.0031
Tyrosine	5.96 × 10^−11^	Higher	0.2799	0.0434
Valine	5.96 × 10^−11^	Higher	0.7959	0.0009
**Carbohydrate**				
Isobar: ribulose 5-phosphate, xylulose 5-phosphate	0.000172	Higher	0.0412	0.0003
Mannose-6-phosphate	1.54 × 10^−7^	Higher	0.6230	0.0005
UDP-glucuronate	5.96 × 10^−11^	Higher	0.0052	0.3599
**Cofactors and vitamins**				
Thiamin (Vitamin B1)	1.54 × 10^−7^	Higher	0.0101	0.0044
**Energy**				
Fumarate	2.08 × 10^−7^	Higher	0.0068	0.0343
Malate	1.63 × 10^−5^	Higher	0.6305	0.1728
Succinate	5.96 × 10^−11^	Higher	0.6842	0.0003
**Lipids**				
1-Arachidonoylglycerophosphoethanolamine	1.13 × 10^−9^	Higher	0.2475	0.1011
1-Docosahexaenoylglycerophosphoethanolamine	3.77 × 10^−5^	Higher	0.9118	0.0434
1-Palmitoylglycerophosphoethanolamine	0.000128	Higher	0.1230	0.0085
1-Palmitoylglycerophosphoinositol	1.16 × 10^−8^	Higher	0.7959	0.4598
1-Stearoylglycerophosphoethanolamine	5.49 × 10^−6^	Higher	0.0892	0.0044
1-Stearoylglycerophosphoinositol	1.24 × 10^−5^	Higher	0.0756	0.2743
2-Arachidonoylglycerophosphoethanolamine	8.28 × 10^−9^	Higher	0.9705	0.0085
2-Docosahexaenoylglycerophosphoethanolamine	1.15 × 10^−5^	Higher	0.0433	0.0031
2-Linoleoylglycerophosphoethanolamine	1.75 × 10^−6^	Higher	0.5966	0.1728
Butyrylcarnitine	4.53 × 10^−6^	Lower	0.2176	0.0003
Deoxycarnitine	0.000023	Lower	0.0003	0.5726
Dihomo-linoleate (20:2n6)	5.96 × 10^−11^	Higher	0.6842	0.2370
Dihomo-linolenate (20:3n3 or n6)	5.96 × 10^−11^	Higher	0.1431	0.0062
Glycerol 3-phosphate (G3P)	5.96 × 10^−11^	Higher	0.5787	0.0266
Linoleate (18:2n6)	5.96 × 10^−11^	Higher	0.6305	0.0831
Mead acid (20:3n9)	5.96 × 10^−11^	Higher	0.2799	0.0021
Myo-inositol	5.96 × 10^−11^	Higher	0.6842	0.0062
Scyllo-inositol	5.96 × 10^−11^	Higher	0.7959	0.6965
**Nucleotide**				
Guanosine 5′- monophosphate (5′-GMP)	6.89 × 10^−7^	Higher	0.0185	0.6334
**Peptide**				
Alanylleucine	1.79 × 10^−9^	Higher	0.3527	0.0003
Glycylglycine	5.96 × 10^−11^	Higher	0.0524	0.2743
Glycylleucine	5.96 × 10^−11^	Higher	0.0007	0.1220
Leucylaspartate	5.96 × 10^−11^	Higher	0.7394	0.0545
Leucylglutamate	2.28 × 10^−7^	Higher	0.2475	0.0005
Leucylglycine	5.78 × 10^−9^	Higher	0.2176	0.0205
Phenylalanylglycine	5.96 × 10^−11^	Higher	0.0005	0.0044
Phenylalanylserine	7.22 × 10^−8^	Higher	0.1051	0.0031
Prolylalanine	2.68 × 10^−9^	Higher	0.5288	0.6334
Prolylglutamate	5.96 × 10^−11^	Higher	0.0068	0.0545
Prolylglycine	5.96 × 10^−11^	Higher	0.2799	0.0676
Serylleucine	3.99 × 10^−9^	Higher	0.1903	0.0031
Seryltyrosine	1.61 × 10^−7^	Higher	0.4359	0.9654
Tyrosylglycine	2.38 × 10^−10^	Higher	0.0029	0.0044
Valylaspartate	9.51 × 10^−8^	Higher	0.0433	0.0031
Valylglycine	7.15 × 10^−10^	Higher	0.0355	0.8968
Valylleucine	1.16 × 10^−8^	Higher	0.3527	0.0014
**Xenobiotics**				
Erythritol	4.07 × 10^−8^	Higher	0.1230	0.0009
**Unknown**				
X – 11,677	8.28 × 10^−9^	Higher	0.0021	0.1728
X − 11,687	5.96 × 10^−11^	Higher	0.0068	0.8968
X − 11,787	2.71 × 10^−5^	Lower	0.3527	0.0831
X − 14,056	8.28 × 10^−9^	Higher	0.4813	0.7618
X − 14,603	4.11 × 10^−6^	Higher	0.0068	0.0067
X − 14,949	7.06 × 10^−5^	Lower	0.0007	0.0545
X − 15,546	5.96 × 10^−11^	Higher	0.0089	0.6334
X − 17,115	1.49 × 10^−7^	Higher	0.0866	0.6965
X − 19,411	1.53 × 10^−7^	Higher	0.2116	0.2370

Sixty-seven metabolites were identified that significantly differentiated colon cancer cell lines from ovarian cancer cell lines according to following criteria: significant difference between ovarian and colon cell lines (p < 2.22 × 10^−4^ for comparison of HCT116 + HCT15 vs. OVCAR3 + SCOV3), but no differences between cell lines of the same origin, (p > 2.22 × 10^−4^ for HCT116 vs. HCT15 and OVCAR3 vs. SCOV3). P values were calculated with metaP server using the Mann–Whitney test.

Metabolites differentiating colon from ovarian cancer cell lines were grouped into different metabolic classes including amino acids, carbohydrates, energy-related metabolites, lipids, nucleotides, dipeptides, and xenobiotics (Table 1). Nine of the 67 metabolites that differentiated ovarian versus colon cancer cell lines were unknown. The majority of metabolites were significantly higher in ovarian cancer cell lines than in colon cancer cell lines (Table 1). In general, ovarian cancer cells displayed significantly higher levels of amino acids, dipeptides (Additional file 6: Figure S3), lipids, and tricarboxylic acid (TCA) cycle intermediates; colon cancer cell lines exhibited higher levels of carnitine and biogenic amines. The metabolites that differentiated colon from ovarian cancer cell lines are highlighted in Figure 2a. They are distributed among the first, second, and fourth clusters.

### Metabolic pathways active in colon and ovarian cancer cell lines

**Figure 3:**
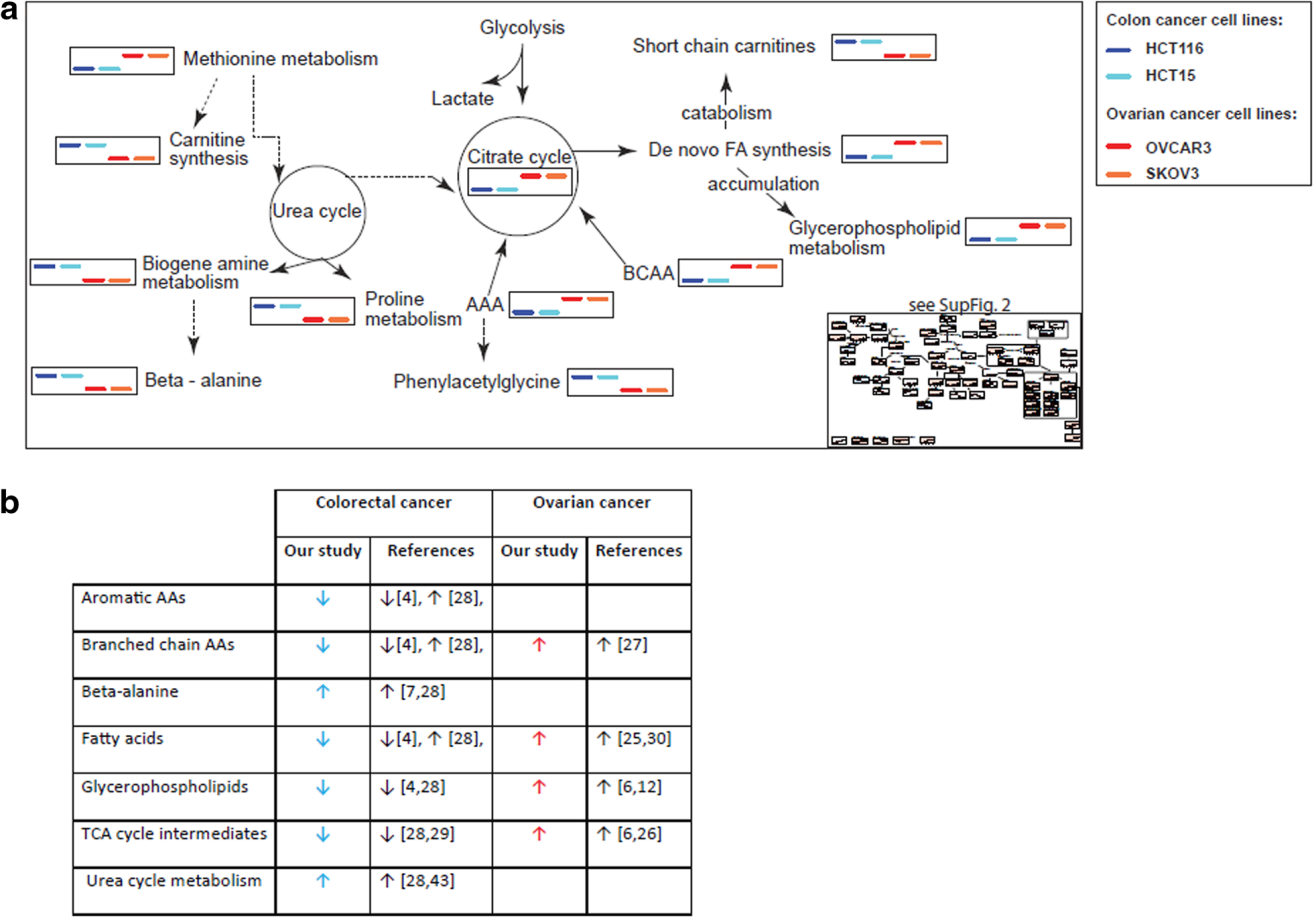
Metabolic diversity in colon and ovarian cancer cell lines. **a** Synthetic view of the metabolic signatures of cell lines derived from colorectal and ovarian cancers. The full data presentation is available in Additional file 7: Figure S4 as *box plots* in a zoomable format. The *blue* color gradation represents colon cancer cell lines (HCT15, *dark blue*; HCT116, *light blue*), *red* and *orange* indicate ovarian cancer cell lines (OVCAR3, *red*; SKOV3, *orange*). **b** Summary of the main findings of this study, as well as previously reported metabolomics changes observed in comparable human biofluids or tissue samples from patients with colon and ovarian carcinomas. *Arrows* indicate direction of changes reported in the literature and in the present study for colon and ovarian cancer cell lines.

We reconstructed a metabolic pathway based on the metabolites that significantly differentiated colon from ovarian cancer cell lines, along with other metabolites identified in the data set that are relevant to these pathways (Additional file 7: Figure S4, and synthesized in Figure 3a).

As presented in the Additional file 7: Figure S4, glucose levels differed strongly between cell lines and were not cancer type-specific. However, glucose-6-phosphate and mannose-6-phosphate levels were higher in both ovarian cancer cell lines, which suggest higher glycolytic activity in comparison to colon cancer cell lines. Lactate was highest in the SKOV3 line, while OVCAR3 exhibited only slight lactate elevation in comparison to colon cancer cell lines.

Levels of TCA cycle intermediates, including succinate, fumarate, and malate, were higher in ovarian cell lines, clearly differentiating them from colon cancer cell lines. The levels of the branched chain amino acids (BCAA) isoleucine and valine and the aromatic amino acids (AAA) tyrosine and phenylalanine were higher in ovarian cancer cell lines. BCAAs can be catabolized to propionyl-CoA, and then to methylmalonyl-CoA and succinyl-CoA, which in turn can be incorporated into the TCA cycle. In addition, phenylalanine and tyrosine can enter the TCA cycle after conversion to fumarate (Figure 3a). Colon cancer cell lines exhibited higher levels of isovalerylcarnitine and isobutyrylcarnitine (degradation products of isoleucine and valine, respectively) than ovarian cancer cells.

Fatty acid (FA) and glycerophospholipid (GPL) signatures strongly differentiated colon from ovarian cancer cell lines, but are similar between cell lines of a shared origin (Additional file 7: Figure S4). FA and GPL levels were significantly lower in colon cancer cell lines, suggesting a release of GPLs from the cellular membrane and further catabolism to FAs. FAs can then be degraded during the process of β-oxidation, which occurs in the mitochondria. Because FAs cannot migrate into the mitochondrial matrix across the mitochondrial membrane alone, long-chain acyl groups bind to carnitines, which serve as a carrier. Accordingly, levels of deoxycarnitine (a carnitine precursor) and other short-chain acylcarnitines were higher in colon cancer cell lines.

Ovarian cancer cell lines exhibit higher levels of FAs and GPLs (seven glycerophosphoethanolamines and two glycerophosphoinositols), which suggests the de novo synthesis and elongation of FAs and GPLs (which requires the acetyl-CoA molecule), as well as their accumulation. Because citrate serves as an acetyl-CoA donor for FA synthesis and elongation [18] and GPLs are composed of FA molecules, higher lipid metabolism might be connected with increased TCA cycle metabolism in ovarian cancer cell lines. Significantly higher levels of linoleate (18:2 n6) and dihomo-γ-linoleate (20:3 n6) in ovarian cancer cell lines might indicate the subsequent desaturation of linoleate (catalyzed by delta-6-desaturase) followed by its further elongation to dihomo-γ-linoleate (catalyzed by elongase-5) (Additional file 7: Figure S4). The ovarian cancer cell lines exhibited elevated methionine metabolism, as shown by significantly higher levels of methionine, *N*-acetyl-methionine, methionine sulfoxide, cystathionine, and dimethylarginine.

Among the urea cycle intermediates, the level of citrulline was higher in ovarian cancer cell lines. Other metabolites connected to the urea cycle were higher in colon cancer- cell lines, such as *N*-acetylputrescine, beta-alanine (both products of putrescine metabolism), and *trans*-4-hydroxyproline (a product of proline metabolism).

## Discussion

Metabolic differences between cancer cells and healthy cells were recognized by Warburg as early as the 1920s [19]. Modern metabolomics tools are able to capture overall metabolite composition in different types of samples. In comparison to healthy cells, the metabolism of cancer cells is generally characterized by elevated catabolism of glucose into lactate, known as the Warburg effect [19], as well as the preferential metabolism of amino acids, including valine, isoleucine, and glutamine [20, 21]. Furthermore, the analysis of 22 diverse tumor types revealed significant heterogeneity in the activity of several metabolic pathways (e.g., oxidative phosphorylation and the TCA cycle) [22], suggesting tumor-specific metabolic differences [23–25]. Thus, metabolic differences among cancers of various origins could be applied to differentiate between primary ovarian cancer and ovarian metastases of colon origin, which have historically been very difficult to distinguish using available radiological and histological approaches. To evaluate this concept, we applied non-targeted metabolomics to four cell lines derived from colon and ovarian cancers.

Among the 225 metabolites analyzed in this study, we found strong metabolic individuality among the different cell lines, with concomitant identity in lines with a shared tissue of origin (colon vs. ovary). To ensure robustness, we normalized metabolic data on cell number as described previously [12, 13]; we excluded normalization against protein content, because it has been reported that this strategy introduces large errors into the data set [26].

We have found 67 metabolites that significantly differentiate colon from ovarian cancer cell lines, supporting the idea that cancers originating from different tissues can be metabolically distinct. These findings are preliminary and cannot be directly translated to clinical applications, and validation in human tissue and biofluids samples is required; however, we compared our data with previous reports comparing healthy controls with colorectal or ovarian cancer patients. Interestingly, our findings were concordant with those of previous studies reporting differences in metabolites and metabolic pathways in biofluids and tissue samples (Figure 3b**)**. For example, previous reports indicate that TCA cycle metabolism, which we observed as lower in colon cancer cell lines, down-regulated in both tissue and plasma samples from patients with colorectal cancer [6, 8, 9, 14, 27–32] (Figure 3b). Similarly, concordant with our analysis of two ovarian cancer cell lines, higher levels of TCA cycle intermediates (including succinate, fumarate, and malate) have been observed in tissue samples from ovarian carcinoma [8, 28].

The TCA cycle is connected to other metabolic pathways, including glycolysis, amino acid catabolism, and fatty acid synthesis; it is a central source of energy for healthy cells. In normal cellular metabolism, glucose is mainly catabolized into pyruvate, which is subsequently converted into acetyl-CoA and enters/supports the TCA cycle. In contrast, cancer cells primarily metaboliz glucose into lactate. Therefore, in cancer cells the TCA cycle is not supported by the glycolytic pathway [33]. Thus the increased level of TCA intermediates in ovarian cancer cell lines suggests both impaired enzyme activity and the supplementation of the TCA cycle in those cell lines with molecules from other pathways (e.g., BCAA and AAA). Notably, alterations in the activity of TCA cycle enzymes, particularly succinate dehydrogenase (SDH) and fumarate hydratase (FH), have been implicated in tumor susceptibility to chemotherapy [34, 35].Accumulated succinate and fumarate have been reported as intracellular messenger molecules that stabilize hypoxia-inducible factor (HIF), which in turn promotes the adaptation of cells to low-oxygen conditions and stimulates angiogenesis [36, 37]. Furthermore, ovarian cancer cell lines exhibited higher levels of BCAA (isoleucine and valine) and AAA (phenylalanine and tyrosine), which can support the TCA cycle at the level of succinate and fumarate, respectively. Elevated BCAA has been reported previously in fluid samples from malignant ovarian cysts [29]. Increased levels of both BCAA and AAA may be linked with alterations in the activity of their transporter, L-type amino acid transporter 1 (LAT1) [38], which has been recognized as an important molecule in the nutrition, proliferation, and migration of ovarian cancer cells [39].

In addition, we observed higher levels of FAs and GPLs in ovarian cancer cell lines. Because the TCA cycle provides building blocks (acetyl-CoA) for FA synthesis and elongation, it may justify the demand of increased TCA cycle metabolism observed in ovarian cancer cell lines. Furthermore, higher phosphatidylethanolamine and phosphatidylcholine levels suggest increases in the de novo synthesis of these GPLs, known as the Kennedy pathway [40]. Our observations are consistent with those of previous studies that reported increased glycerophospholipids as a signature of ovarian cancer (Figure 3b) [8, 14]. For instance, elevated phosphocholine is concordant with the previously reported enrichment of phosphocholine in tissue samples from ovarian cancer patients [14]. Phosphatidylethanolamine can serve as a substrate for phosphatidylcholines, the extreme elevation of which has been reported previously in ovarian carcinomas [14]. Alteration in GPLs may be connected with their roles in membrane integrity and mitogenic signal transduction [41]. Furthermore, increased phosphatidylinositol has been associated with the deregulation of the phosphoinositide-3 kinase (PI3K) pathway, which in turn promotes carcinogenesis and angiogenesis [42].

Colon cancer cell lines displayed higher levels of beta-alanine and *N*-acetylputrescine. Significant elevation of the metabolite *N*-acetylputrescine has been reported previously in colorectal cancer tissue [9, 30]. A biogenic amine, *N*-acetylputrescine may be associated with urea cycle metabolism. Ornithine decarboxylase (ODC) is a first and rate-limiting factor in polyamine biosynthesis; it catalyzes the conversion of ornithine to putrescine [43], which is subsequently metabolized into *N*-acetylputrescine. Increased ODC activity in colorectal cancer has been reported [44]. Our observation of increased urea cycle rate and biogenic amines, as well as lower level of TCA cycle intermediates, is consistent with previous reports on tissue and biofluid samples from colon cancer patients [6, 30, 31, 45] (Figure 3b).

Lower levels of BCAA and AAA observed in colon cancer cell lines may be supported by a previous LC/MS-based study that reported decreased plasma amino acids in patients with colorectal cancer [6]. However, a GC/MS-based study of tissue samples from colorectal cancer reported increases in amino acid levels [30]. The different technologies used might explain the discrepancies between the different studies. However, BCAA catabolism may be associated with isovalerylcarnitine and isobutyrylcarnitine metabolism [46]; higher levels of both molecules were identified in colon cancer-derived cell lines, which further supports our findings.

The observation of significantly lower levels of GPLs and FAs in colon cancer cell lines is consistent with previous studies in human plasma [6] and tissue samples [30] (Figure 3b). Overall our observations suggest that in colon cancer cell lines, GPLs are hydrolyzed into FAs, which are then catabolized. The primary pathway for FAcatabolism is mitochondrial β-oxidation [47]. However, the mitochondrial membrane is impermeable to free FAs, FA transport across the mitochondrial membrane requires a carnitine shuttle [47]. Accordingly, colon cancer cell lines exhibit higher levels of deoxy-carnitine, an intermediate in carnitine synthesis [48]. Colon cancer cell lines also exhibit higher levels of butyrylcarnitine and acylcarnitine, which may be associated with increased β-oxidation [49]. Notably, by undergoing beta-oxidation, FAs produce twice as much ATP as carbohydrates, which strongly support cancer function [50].

## Conclusions and future directions

The main goal of this study was to examine metabolic diversity between colon and ovarian cancer cell lines. After further evaluation in clinical samples, this information might enable clinicians to distinguish primary ovarian cancer from ovarian metastases of colorectal origin. Our study revealed 67 metabolites that significantly differentiated colorectal from ovarian cancer cell lines, and may potentially be useful in cancer screening and diagnosis. Additionally, in this study we highlighted four pathways (out of hundreds) that should be extensively examined. The ovarian cancer cell lines were mainly characterized by elevated TCA cycle intermediates and elevated lipid synthesis. This information might assist novel drug design or drug repurposing, focusing on enzymes/transporters involved in lipid and TCA cycle metabolism. In turn, the metabolic signatures of colon cancer cell lines suggest increased β-oxidation and increased urea cycle activity, which may indicate a demand for the removal of excess ammonia after rapid amino acid catabolism. These observations suggest that targeting β-oxidation and/or urea cycle metabolism in colon cancer might be successful strategies to control this neoplastic disease. Although there are incontestable benefits to the use of cell culture as a model system for metabolomics studies [10], additional investigations that employ primary cells and samples from malignant tissue and human biofluids are needed in order to validate and further refine the signatures reported in this study.

## Additional files

Additional file 1:
**Supplemental Figure 1.** Identification of outliers using PCA. PCA score plots generated by the *meta*P server reveal distinct clustering of HCT15 (*dark blue*), HCT116 (*light blue*), OVCAR3 (*red*), and SKOV3 (*orange*). Two sample outliers were identified in the SKOV3 cell line and were removed from the analysis. Additional file 2:
**Supplemental Table 1.** Metabolites present in all examined cell lines. Star (*) indicates compounds that have not been officially “plexed” (based on a standard), although we are confident in their identity. Additional file 3:
**Supplemental Figure 2.** Examples of cell line-specific metabolic signatures. The log-scaled metabolite intensities are presented as box plots (arbitrary units suppressed). P-values calculated using the Kruskal–Wallis test are shown in each diagram; **” 1% significance after Bonferroni correction; * 5% significance after Bonferroni correction. Full access to all plots is available via the *meta*P server: http://metap.helmholtz-muenchen.de/metap3/run.cgi?ID=141536220398464. Additional file 4:
**Supplemental Table 2.** Metabolites that significantly distinguish SKOV3 from OVCAR3. Additional file 5:
**Supplemental Table 3.** Metabolites that significantly distinguish HCT116 from HCT15. Additional file 6:
**Supplemental Figure 3.** Dipeptides were significantly upregulated in ovarian cancer cells compared with colon cancer cells. The log-scaled metabolite intensities are presented as box plots representing the median values of experiments performed in 10 (HCT15, HTC116, OVCAR3) and 8 (SKOV3) replicates. Values were obtained after statistical data analysis using the *meta*P server. Additional file 7:
**Supplemental Figure 4.** Metabolic pathway reconstructed from the metabolic data for colon and ovarian cancer cell lines. The log-scaled metabolite intensities are presented as box plots representing the median values of experiments performed in 10 (HCT15, HTC116, OVCAR3) and 8 (SKOV3) replicates. Values were obtained after statistical data analysis using the *meta*P server. Metabolites highlighted in color (blue or red) indicate molecules that significantly differentiated colon from ovarian cancers. Blue reflects metabolites observed at significantly higher levels in colon cancer cell lines; red reflects metabolites observed at significantly higher levels in ovarian cancer cell lines. Δ6D: delta-6-desaturase; EL5: elongase.
