# Supplementary figures and images for: Metabolic signatures differentiate ovarian from colon cancer cell lines
Source: J Transl Med. 2015 Jul 14;13:223. doi: 10.1186/s12967-015-0576-z (PMC4499939; doi:10.1186/s12967-015-0576-z)

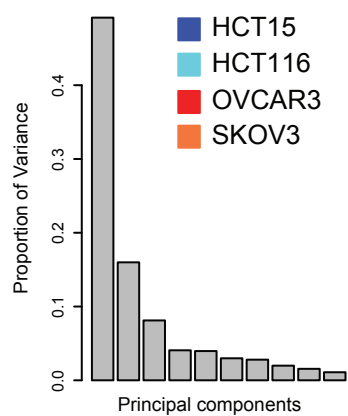

Legend:

- HCT15 (Blue)
- HCT116 (Cyan)
- OVCAR3 (Red)
- SKOV3 (Orange)

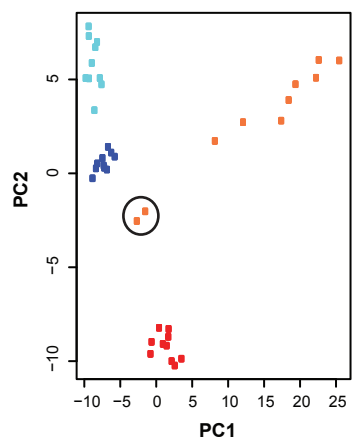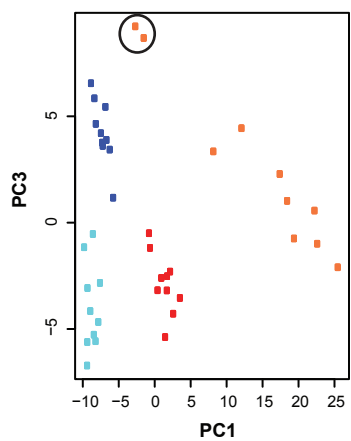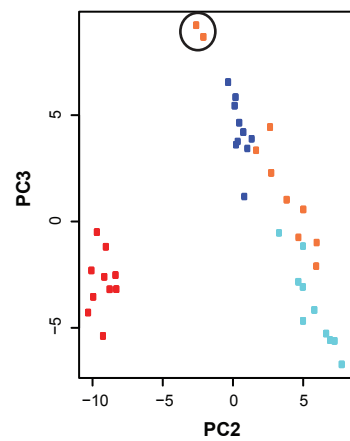

Supplement: Additional file 1: — Supplemental Figure 1. Identification of outliers using PCA. PCA score plots generated by the metaP server reveal distinct clustering of HCT15 (dark blue), HCT116 (light blue), OVCAR3 (red), and SKOV3 (orange). Two sample outliers were identified in the SKOV3 cell line and were removed from the analysis. [file 12967_2015_576_MOESM1_ESM.pdf]

A)

X - 13505

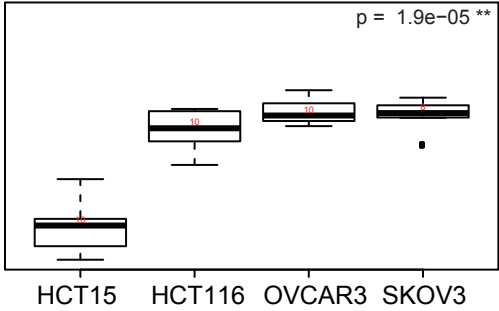

B)

4-guanidinobutanoate

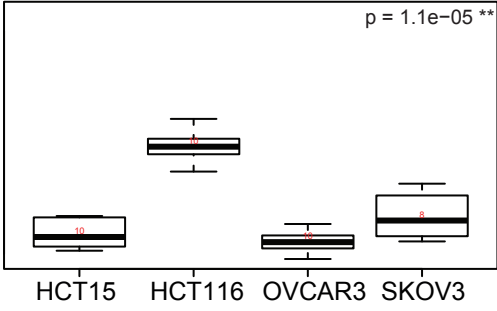

C)

Cytidine

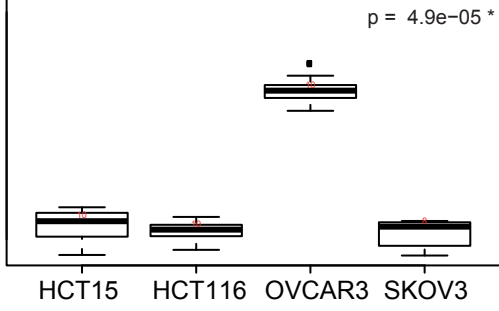

D)

Tryptophan

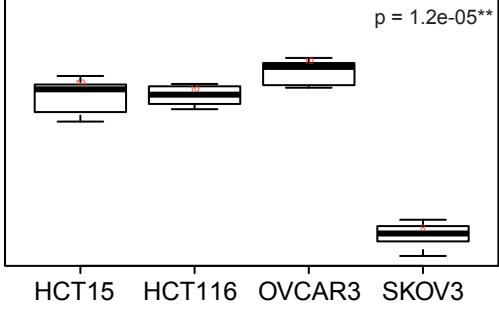

Kynurenine

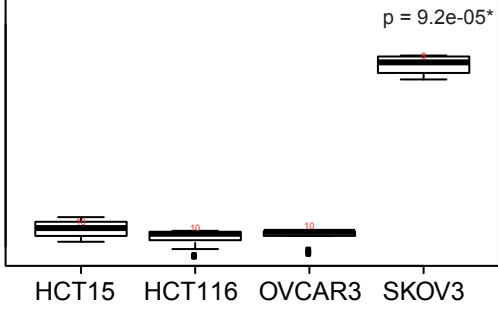

Glutamine

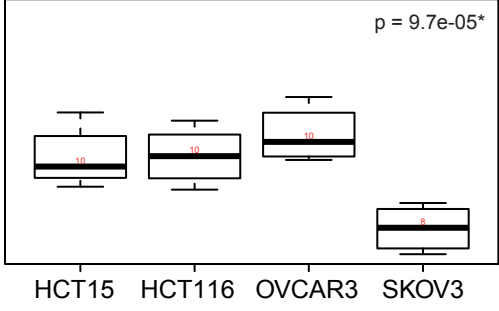

Glutamate

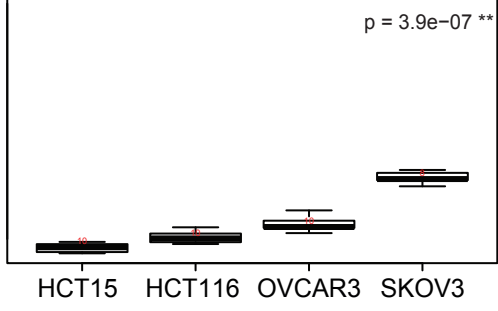

Supplement: Additional file 3: — Supplemental Figure 2. Examples of cell line-specific metabolic signatures. The log-scaled metabolite intensities are presented as box plots (arbitrary units suppressed). P-values calculated using the Kruskal–Wallis test are shown in each diagram; **” 1% significance after Bonferroni correction; * 5% significance after Bonferroni correction. Full access to all plots is available via the metaP server: http://metap.helmholtz-muenchen.de/metap3/run.cgi?ID=141536220398464. [file 12967_2015_576_MOESM3_ESM.pdf]

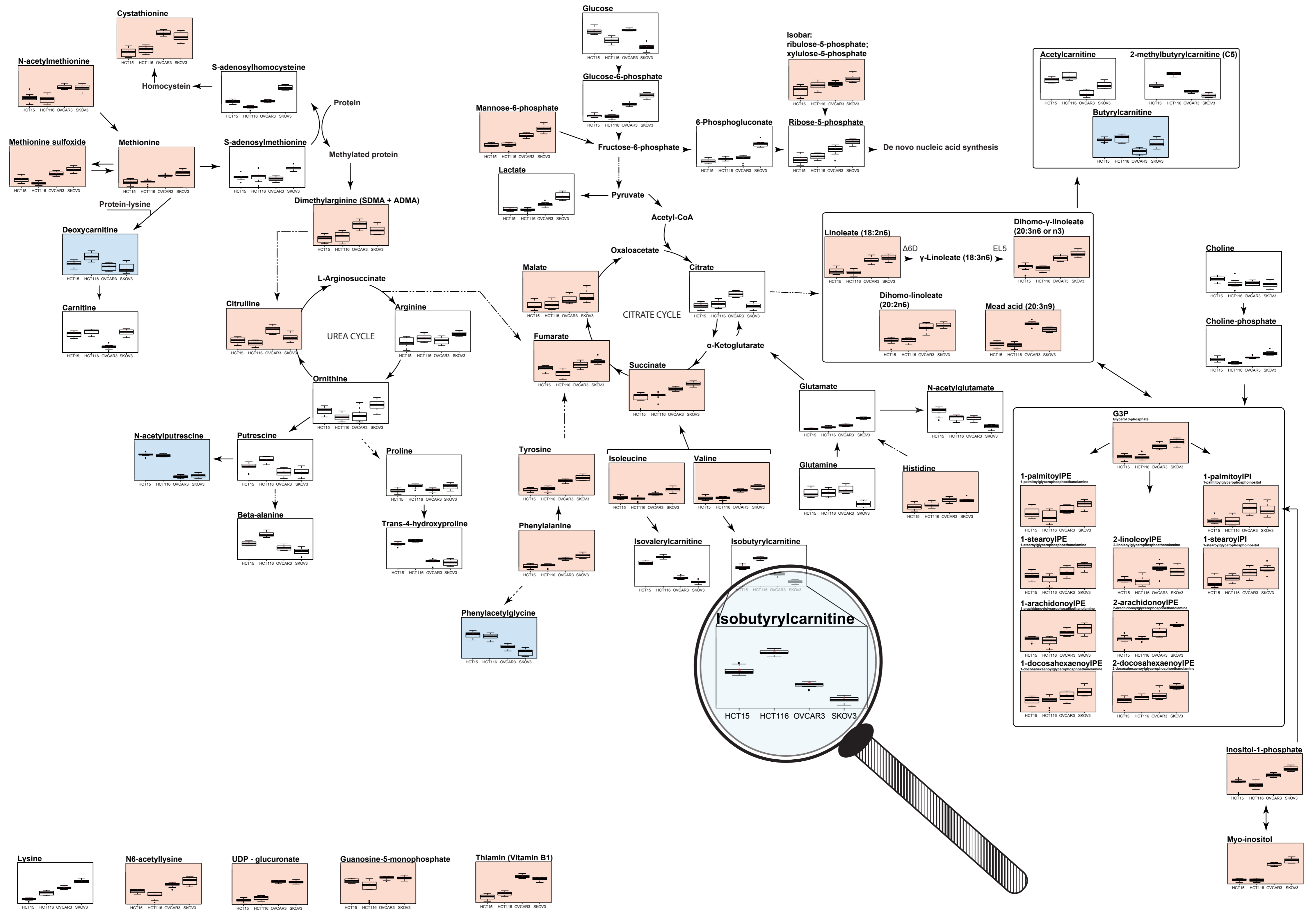

Supplement: Additional file 6: — Supplemental Figure 3. Dipeptides were significantly upregulated in ovarian cancer cells compared with colon cancer cells. The log-scaled metabolite intensities are presented as box plots representing the median values of experiments performed in 10 (HCT15, HTC116, OVCAR3) and 8 (SKOV3) replicates. Values were obtained after statistical data analysis using the metaP server. [file 12967_2015_576_MOESM6_ESM.pdf]
