# Supplementary material for: Metabolic signatures differentiate ovarian from colon cancer cell lines
Source: J Transl Med. 2015 Jul 14;13:223. doi: 10.1186/s12967-015-0576-z (PMC4499939; doi:10.1186/s12967-015-0576-z)
Supplement: Additional file 4: — Supplemental Table 2. Metabolites that significantly distinguish SKOV3 from OVCAR3. [file 12967_2015_576_MOESM4_ESM.docx]

| Metabolite name | P-value | Level in SKOV3 |
| --- | --- | --- |
| 1 - oleoylplasmenylethanolamine | 4.57E-05 | up |
| 1 - palmitoylglycerophosphocholine | 4.57E-05 | up |
| 1 - palmitoylplasmenylethanolamine. | 4.57E-05 | up |
| 10 - nonadecenoate | 4.57E-05 | up |
| 2 - aminoadipate | 4.57E-05 | up |
| 3 - (4 - hydroxyphenyl) lactate | 4.57E-05 | down |
| 5 - oxoproline | 4.57E-05 | up |
| 6 - phosphogluconate | 4.57E-05 | up |
| Acetylcarnitine | 4.57E-05 | up |
| Adenine | 4.57E-05 | up |
| Adenosine 5 - diphosphate (ADP) | 4.57E-05 | up |
| Arachidonate (20:4n6) | 4.57E-05 | up |
| Aspartylaspartate | 4.57E-05 | up |
| Aspartylleucine | 4.57E-05 | up |
| Carnitine | 4.57E-05 | up |
| Cholesterol | 4.57E-05 | up |
| Cis - vaccenate (18:1n7) | 4.57E-05 | up |
| Citrate | 4.57E-05 | down |
| Creatine | 4.57E-05 | down |
| Creatinine | 4.57E-05 | down |
| Cysteinylglycine | 4.57E-05 | up |
| Cytidine 5 - diphosphocholine | 4.57E-05 | up |
| Cytidine diphosphate | 4.57E-05 | up |
| Cytidine 5' - diphosphoethanolamine | 4.57E-05 | up |
| Docosadienoate (22:2n6) | 4.57E-05 | up |
| Docosahexaenoate (DHA 22:6n3) | 4.57E-05 | up |
| Docosapentaenoate (DPA 22:5n3) | 4.57E-05 | up |
| Eicosapentaenoate (EPA 20:5n3) | 4.57E-05 | up |
| Eicosenoate (20:1n9 or n11) | 4.57E-05 | up |
| Gamma - glutamylglutamate | 4.57E-05 | up |
| Gamma - glutamylisoleucine | 4.57E-05 | up |
| Gamma - glutamylleucine | 4.57E-05 | up |
| Gamma - glutamylthreonine | 4.57E-05 | up |
| Glucose | 4.57E-05 | down |
| Glucose - 6 - phosphate (G6P) | 4.57E-05 | up |
| Glutamate | 4.57E-05 | up |
| Glutamine | 4.57E-05 | down |
| Glutathione , oxidized (GSSG) | 4.57E-05 | up |
| Glutathione , reduced (GSH) | 4.57E-05 | up |
| Glycerophosphoethanolamine | 4.57E-05 | up |
| Glycerophosphorylcholine (GPC) | 4.57E-05 | up |
| Glycylproline | 4.57E-05 | up |
| Guanosine 5' - diphosphate (GDP) | 4.57E-05 | up |
| Hexanoylcarnitine | 4.57E-05 | up |
| Homostachydrine | 4.57E-05 | down |
| Inositol 1 - phosphate (I1P) | 4.57E-05 | up |
| Isobutyrylcarnitine | 4.57E-05 | down |
| Isoleucylglycine | 4.57E-05 | up |
| Lactose | 4.57E-05 | up |
| Leucine | 4.57E-05 | up |
| Linolenate [alpha or gamma (18:3n3 or n6) | 4.57E-05 | down |
| Lysine | 4.57E-05 | up |
| N - acetylaspartate (NAA) | 4.57E-05 | down |
| N - acetylglutamate | 4.57E-05 | down |
| N - acetylneuraminate | 4.57E-05 | up |
| N - acetylthreonine | 4.57E-05 | down |
| Nicotinamide adenine dinucleotide (NAD) | 4.57E-05 | up |
| Oleate (18:1n9) | 4.57E-05 | up |
| Oleic ethanolamide | 4.57E-05 | up |
| Palmitoleate (16:1n7) | 4.57E-05 | down |
| Palmitoyl sphingomyelin | 4.57E-05 | up |
| Phenol red | 4.57E-05 | down |
| Pyridoxine (Vitamin B6) | 4.57E-05 | down |
| Pyroglutamine. | 4.57E-05 | down |
| S - adenosylhomocysteine (SAH) | 4.57E-05 | up |
| S - adenosylmethionine (SAM) | 4.57E-05 | up |
| Sorbitol | 4.57E-05 | up |
| Threonine | 4.57E-05 | down |
| Threonylleucine | 4.57E-05 | up |
| Tryptophan | 4.57E-05 | down |
| Tryptophylglutamate | 4.57E-05 | up |
| Tyrosylglutamate | 4.57E-05 | up |
| Uracil | 4.57E-05 | up |
| Uridine 5' - diphosphate (UDP) | 4.57E-05 | up |
| X - 10445 | 4.57E-05 | down |
| X - 11583 | 4.57E-05 | up |
| X - 12855 | 4.57E-05 | up |
| X - 13230 | 4.57E-05 | up |
| X - 13512 | 4.57E-05 | up |
| X - 14568 | 4.57E-05 | down |
| X - 14577 | 4.57E-05 | up |
| X - 15117 | 4.57E-05 | up |
| X - 15375 | 4.57E-05 | up |
| X - 15382 | 4.57E-05 | up |
| X - 15484 | 4.57E-05 | up |
| X - 15564 | 4.57E-05 | up |
| X - 15680 | 4.57E-05 | up |
| 1 - palmitoleoylglycerophosphoethanolamine. | 9.14E-05 | down |
| 5 - methylthioadenosine (MTA) | 9.14E-05 | up |
| Isobar: fructose 1,6 - diphosphate; glucose 1,6 -diphosphate; myo - inositol 14 or 13 diphosphate | 9.14E-05 | up |
| Isobar: UDP - acetylglucosamine; UDP - acetylgalactosamine | 9.14E-05 | up |
| Lactate | 9.14E-05 | up |
| N - acetylalanine | 9.14E-05 | down |
| N - formylmethionine | 9.14E-05 | up |
| Phenylalanylalanine | 9.14E-05 | up |
| 10 - heptadecenoate (17:1n7) | 1.83E-04 | up |
| 2 - methylbutyrylcarnitine | 1.83E-04 | down |
| Adenosine 5' - monophosphate (AMP) | 1.83E-04 | up |
| Aarginine | 1.83E-04 | up |
| Choline phosphate | 1.83E-04 | up |
| Isovalerylcarnitine | 1.83E-04 | down |
| Methylphosphate | 1.83E-04 | up |
| Ribose 5 - phosphate | 1.83E-04 | up |
| Uridine monophosphate (5' or 3') | 1.83E-04 | up |
| X - 11381 | 1.83E-04 | up |

**Supplemental Table 2** Metabolites that significantly distinguish SKOV3 from OVCAR3
