# Supplementary material for: Metabolic signatures differentiate ovarian from colon cancer cell lines
Source: J Transl Med. 2015 Jul 14;13:223. doi: 10.1186/s12967-015-0576-z (PMC4499939; doi:10.1186/s12967-015-0576-z)
Supplement: Additional file 5: — Supplemental Table 3. Metabolites that significantly distinguish HCT116 from HCT15. [file 12967_2015_576_MOESM5_ESM.docx]

| Metabolite name | P-value | Level in HCT116 |
| --- | --- | --- |
| 1 - palmitoylglycerophosphocholine | 1.08E-05 | up |
| 2 - aminoadipate | 1.08E-05 | up |
| 2 - methylbutyrylcarnitine | 1.08E-05 | up |
| 4 - guanidinobutanoate | 1.08E-05 | up |
| Adenosine - 5 - monophosphate (AMP) | 1.08E-05 | down |
| Beta alanine | 1.08E-05 | up |
| Cytidine diphosphate | 1.08E-05 | down |
| Glucose | 1.08E-05 | down |
| Glutathione reduced (GSH) | 1.08E-05 | down |
| Homostachydrine | 1.08E-05 | up |
| Isobutyrylcarnitine | 1.08E-05 | up |
| Isovalerylcarnitine | 1.08E-05 | up |
| Lysine | 1.08E-05 | up |
| Ophthalmate | 1.08E-05 | up |
| Phenol red | 1.08E-05 | down |
| Phosphate | 1.08E-05 | down |
| Pro-hydroxy-pro | 1.08E-05 | up |
| Pyroglutamine | 1.08E-05 | up |
| S - adenosylhomocysteine (SAH) | 1.08E-05 | down |
| Spermidine | 1.08E-05 | down |
| Stachydrine | 1.08E-05 | up |
| Uridine 5 - diphosphate (UDP) | 1.08E-05 | down |
| Xanthine | 1.08E-05 | up |
| X - 13505 | 1.08E-05 | up |
| X - 14568 | 1.08E-05 | down |
| X - 14577 | 1.08E-05 | down |
| X - 15117 | 1.08E-05 | up |
| X - 15192 | 1.08E-05 | up |
| X - 20080 | 1.08E-05 | down |
| Adenine | 2.17E-05 | down |
| Adenosine | 2.17E-05 | down |
| Choline phosphate | 2.17E-05 | down |
| Gamma glutamylisoleucine | 2.17E-05 | down |
| Glycerol | 2.17E-05 | down |
| Isobar: UDP - acetylglucosamine; UDP - acetylgalactosamine | 2.17E-05 | up |
| Pyridoxine (Vitamin B6) | 2.17E-05 | down |
| X - 13230 | 2.17E-05 | up |
| Aspartate | 4.33E-05 | up |
| N - acetylaspartate (NAA) | 4.33E-05 | down |
| Pantothenate | 4.33E-05 | down |
| Sorbitol | 4.33E-05 | down |
| Trans - 4 - hydroxyproline | 4.33E-05 | up |
| Cytidine 5 - monophosphate (5 - CMP) | 7.58E-05 | down |
| 5 - methylthioadenosine (MTA) | 0.000129901 | down |
| Cytidine 5 - diphosphocholine | 0.000129901 | down |
| Glycylproline | 0.000129901 | up |
| N - acetylthreonine | 0.000129901 | up |
| Glycerophosphoethanolamine | 0.000178614 | up |

**Supplemental Table 3** Metabolites that significantly distinguish HCT116 from HCT15
