# Supplementary material for: Metabolic signatures differentiate ovarian from colon cancer cell lines
Source: J Transl Med. 2015 Jul 14;13:223. doi: 10.1186/s12967-015-0576-z (PMC4499939; doi:10.1186/s12967-015-0576-z)
Supplement: Additional file 7: — Supplemental Figure 4. Metabolic pathway reconstructed from the metabolic data for colon and ovarian cancer cell lines. The log-scaled metabolite intensities are presented as box plots representing the median values of experiments performed in 10 (HCT15, HTC116, OVCAR3) and 8 (SKOV3) replicates. Values were obtained after statistical data analysis using the metaP server. Metabolites highlighted in color (blue or red) indicate molecules that significantly differentiated colon from ovarian cancers. Blue reflects metabolites observed at significantly higher levels in colon cancer cell lines; red reflects metabolites observed at significantly higher levels in ovarian cancer cell lines. Δ6D: delta-6-desaturase; EL5: elongase. [file 12967_2015_576_MOESM7_ESM.pdf]

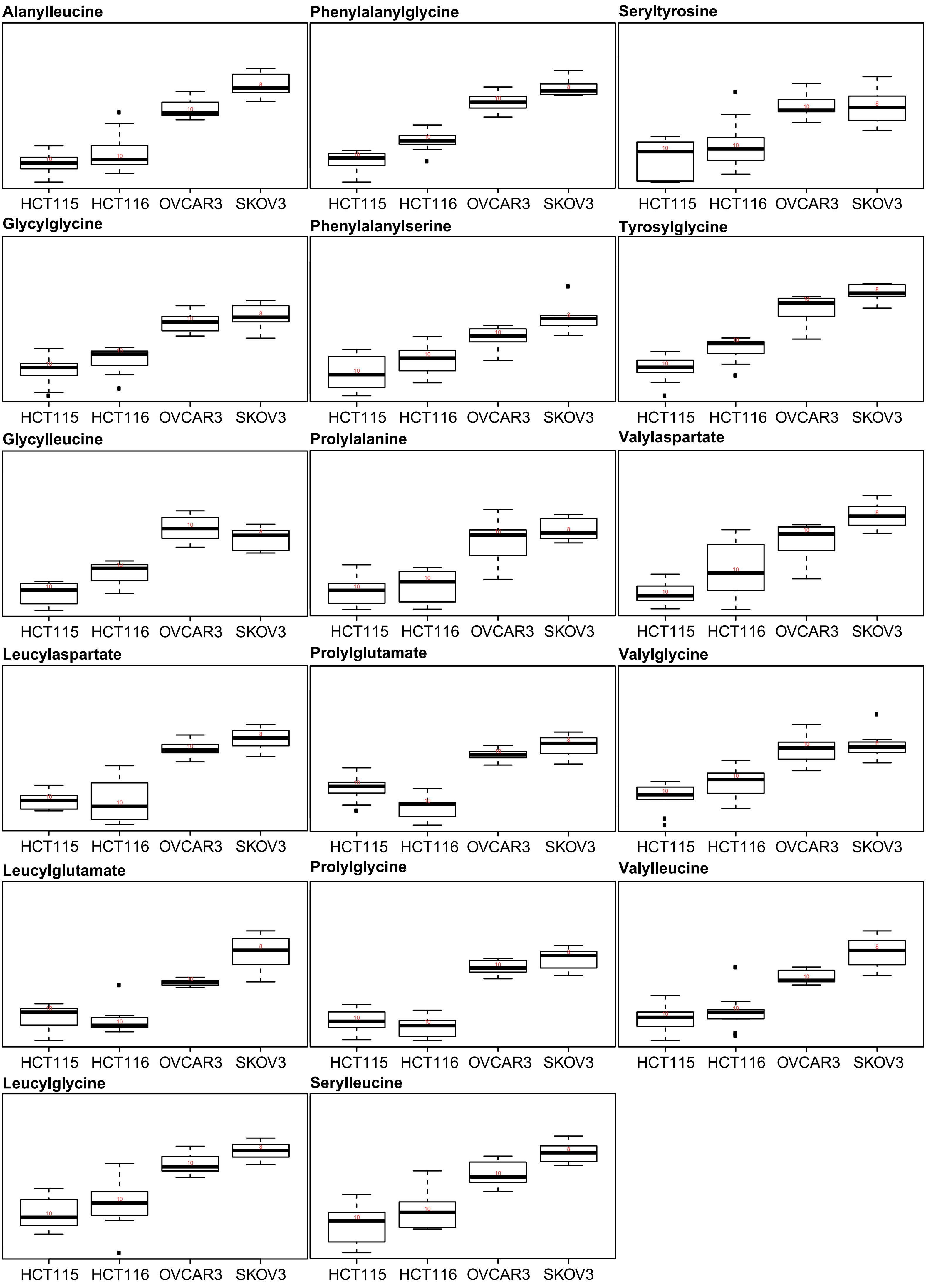
Alanylleucine
HCT115 HCT116 OVCAR3 SKOV3
Phenylalanylglycine
HCT115 HCT116 OVCAR3 SKOV3
Seryltyrosine
HCT115 HCT116 OVCAR3 SKOV3
Glycylglycine
HCT115 HCT116 OVCAR3 SKOV3
Phenylalanylserine
HCT115 HCT116 OVCAR3 SKOV3
Tyrosylglycine
HCT115 HCT116 OVCAR3 SKOV3
Glycylleucine
HCT115 HCT116 OVCAR3 SKOV3
Prolylalanine
HCT115 HCT116 OVCAR3 SKOV3
Valylaspartate
HCT115 HCT116 OVCAR3 SKOV3
Leucylaspartate
HCT115 HCT116 OVCAR3 SKOV3
Prolylglutamate
HCT115 HCT116 OVCAR3 SKOV3
Valylglycine
HCT115 HCT116 OVCAR3 SKOV3
Leucylglutamate
HCT115 HCT116 OVCAR3 SKOV3
Prolylglycine
HCT115 HCT116 OVCAR3 SKOV3
Valylleucine
HCT115 HCT116 OVCAR3 SKOV3
Leucylglycine
HCT115 HCT116 OVCAR3 SKOV3
Serylleucine
HCT115 HCT116 OVCAR3 SKOV3
